# Application of Extended Reality (Virtual Reality and Mixed Reality) Technology in Laparoscopic Liver Resections

**DOI:** 10.7759/cureus.44520

**Published:** 2023-09-01

**Authors:** Shigetoshi Naito, Masatoshi Kajiwara, Ryo Nakashima, Takahide Sasaki, Suguru Hasegawa

**Affiliations:** 1 Gastroenterological Surgery, Fukuoka University Hospital, Fukuoka, JPN; 2 Gastroenterological Surgery, Fukuoka University Faculty of Medicine, Fukuoka, JPN

**Keywords:** computer simulation, mixed reality, virtual reality, hepatectomy, laparoscopic surgery

## Abstract

Background and purpose

Laparoscopic liver resection (LLR) has recently gained popularity owing to advances in surgical techniques. Difficulties in LLR may be influenced by anatomical factors. This study presents a comprehensive overview of LLR performed using extended reality (XR) technology.

Methods

Six patients underwent LLR performed wearing HoloLens2® XR (Microsoft Corporation, Redmond, Washington, United States) technology. We performed dynamic contrast-enhanced CT scans before surgery and used the data to construct three-dimensional images.

Results

Of the six patients, two were diagnosed with colorectal liver metastases, two with hepatocellular carcinoma, and one with intrahepatic cholangiocarcinoma. The median maximum tumor diameter was 31 mm (range, 23-80 mm). One patient had liver cirrhosis, with Child-Pugh classification grade B. Anatomical resection was performed in three patients (60%), with a median difficulty score of 7 (intermediate). No conversions to open surgery were necessary. The median operative time and estimated blood loss were 444 minutes (range, 337-597 minutes) and 200 mL (range, 100-1000 mL), respectively. Postoperative complications (Clavien-Dindo classification grade II) were observed in one patient. All six cases achieved negative surgical margins.

Conclusions

LLR using XR technology enhances surgical visualization and anatomical recognition. The incorporation of XR technology into LLR offers advantages over traditional two-dimensional imaging.

## Introduction

In recent years, laparoscopic surgery has gained popularity due to advances in surgical techniques. It has also demonstrated promising outcomes in laparoscopic liver resection (LLR) [1-3]. However, the difficulty of LLR may be influenced by anatomical factors and other considerations, necessitating a thorough examination of the indications for intervention. Recently, it has become increasing common to adopt three-dimensional (3D) models created using preoperative images to gain an improved understanding of surgical anatomy [4].

Ultrasonography is also commonly used during surgery to locate deep vascular structures. However, it is important to note that the images obtained from these examinations are viewed using two-dimensional monitors, which may introduce variations when representing the 3D anatomy of the human body. Extended reality (XR) is a general term for image processing technology that creates a space that provides a simulated experience by fusing real and virtual worlds, such as virtual reality (VR), augmented reality (AR), and mixed reality (MR) [5]. VR is a computer-generated simulation that uses special electronic equipment to represent a series of images and sounds depicting real places or situations, allowing users to interact with them in a seemingly real or physical manner. Using a headset, VR can transmit visual and auditory information, as well as various sensations, creating a sense of being in a virtual or imagined environment [6]. The closely related AR uses elements of VR and superimposes them onto a real-world environment in the form of a live video displayed on the screen of an electronic device [7]. MR is a novel technology that has gained increased attention, as it overcomes the limitations of AR, such as the inability to interact with 3D data packets, and the limitations of VR, which exclude the real-world environment. MR is a hybrid of AR and VR in which real and virtual images are intertwined, allowing interaction and manipulation in both real and virtual environments [5]. These technologies have been used in several medical fields [8].

Therefore, we applied VR and MR technologies to laparoscopic liver surgery. XR technology enables us to provide surgical support through stereoscopic vision and virtual environments [9]. In our department, we introduced a system called Holoeyes MD® from Holoeyes, Inc. (Tokyo, Japan), which converts 3D reconstructed images into polygon models based on preoperative image data and enables stereoscopic viewing using VR technology. Additionally, we incorporated MR technology during surgery, projecting navigational images within the surgeon's field of view with 3D visualization and anatomical recognition. Here, we provide a comprehensive overview of LLR performed with VR and illustrate how this technology may contribute to surgical safety.

## Materials and methods

Six LLR procedures were performed using XR technology between September 2022 and May 2023 at Fukuoka University Hospital, Fukuoka, Japan. The study was approved by the Ethics Committee of Fukuoka University Hospital (approval number: U18-12-003), and informed consent was obtained in writing from the patients. Perioperative parameters, such as patient age, sex, BMI, target disease, tumor size, tumor location, difficulty score for LLR, operative procedure, operative time, estimated blood loss, and length of postoperative hospital stay, were retrospectively obtained from medical records [10]. All continuous variables are expressed as medians with ranges. Postoperative complications are expressed using the Clavien-Dindo classification [11].

Pre-/perioperative management

**Figure 1:**
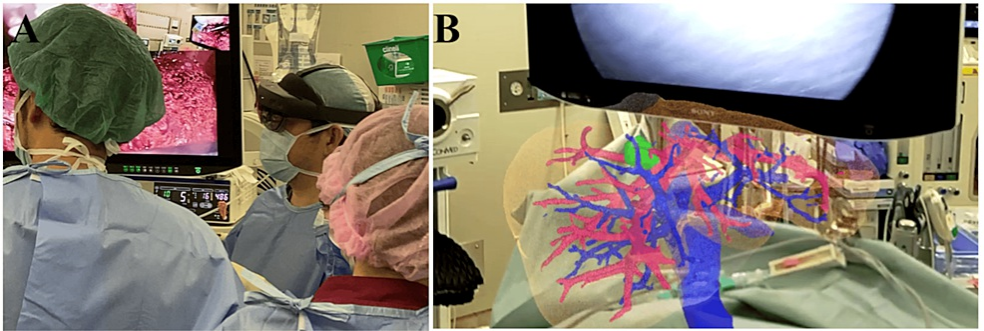
Surgical intervention while wearing the HoloLens2* A: The surgeon performs the surgery while wearing the HoloLens2. It is transparent and allows the surgeon to perform the surgery without obstructing the field of view. B: The image as seen by the surgeon. Holograms appear in front of their eyes, allowing the surgeon to compare the hologram with the images from the endoscope. *Microsoft Corporation, Redmond, Washington, United States

We performed dynamic contrast-enhanced computed tomography (CECT) scans before surgery and used these data to construct 3D images, which were created using Ziostation2® (Ziosoft, Inc. Tokyo, Japan). Cases in which CECT could not be performed due to allergies or renal dysfunction were not included in the XR analysis. After converting the 3D data into polygon data, the data were uploaded to the Holoeyes MD service website. Within approximately 15 minutes, the system automatically generated VR data, called holograms. Furthermore, the data could be downloaded to commercial VR devices, such as Meta Quest 2® (Meta Platforms, Inc., Menlo Park, California, United States) and HoloLens2® (Microsoft Corporation, Redmond, Washington, United States). The HoloLens is an optical see-through (OST) head-mounted display (HMD) that enables the projection of virtual content onto the user’s real-world field of vision [12]. Weighing approximately 579 g, the HoloLens provides a unique MR experience by seamlessly merging virtual and physical environments. The HoloLens2 was worn during the surgery, and liver resection was performed using the MR technology. The holograms were then checked whenever a better understanding of the anatomy was required (Figure 1).

Surgical technique: a representative case

**Figure 2:**
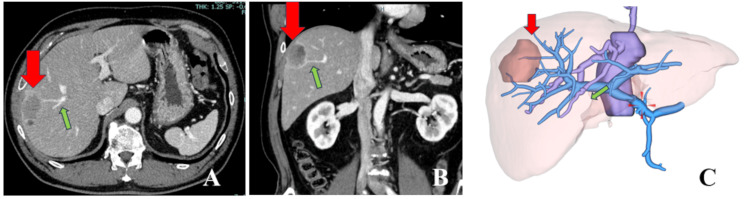
Preoperative images A and B: Findings of preoperative contrast-enhanced computed tomography. C: 3D construction of the liver (red arrow heads indicate the tumor; green arrows indicate the G8 branches). The tumor measured 42 mm and was located in segment 8 of the liver. It extended deep within the liver, and at a distance of 1 cm from the deepest part, a branch of the portal vein (G8) was present. The plan was to perform a liver resection targeting this area.

An LLR was planned for Case 5 with metastatic liver cancer located in segment 8. The tumor was 42 mm in size and had invaded deep into the liver. Preoperative CECT images revealed the presence of a G8 branch at the location where a 1-cm margin was required. Therefore, LLR was planned to resect the G8 branch (Figure 2).

Before surgery, VR was used to visualize the resection process. We observed the potential exposure of the Glisson and vein branches along the path. Additionally, we intuitively confirmed the positioning and movement of the liver in its passive state through interactive manipulation in a VR environment (Video 1).

**Video 1:** Video of preoperative simulation in virtual reality (VR) space

During actual surgery, we wore the HoloLens 2, which allowed us to view holograms overlaid on our field of vision while performing the dissection. As holograms are virtual and not physical images, they provide a clean sterile environment during the surgical intervention. On encountering the Glisson branches, we compared the holograms with the surrounding anatomy to confirm whether they were the target branches. Once we determined that they were indeed the intended Glisson branches, we proceeded with the dissection, completing the liver resection (Video 2).

**Video 2:** Gleason treatment during laparoscopic liver S8 resection The right side is shown from the perspective of the operating surgeon, whereas the left side is footage from the endoscopic camera.

**Figure 3:**
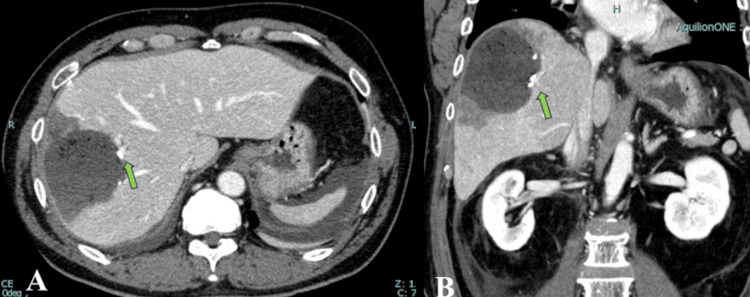
Postoperative images A and B postoperative contrast-enhanced computed tomography findings (green arrows indicate the root of G8 branches). In accordance with the surgical plan, we confirmed that we had successfully resected the root of the G8 branches.

CT was performed on the postoperative day after surgery. This confirmed that the target Glisson branch was successfully addressed while preserving the other G8 branches (Figure 3).

## Results

Table 1 presents the characteristics of all the patients included in the study. The median age of the six patients was 61 years (range, 25-76 years). Of these, three patients were diagnosed with colorectal liver metastasis, two with hepatocellular carcinoma, and one with intrahepatic cholangiocarcinoma. The median maximum tumor diameter was 30 mm (range, 14-80 mm). One patient had liver cirrhosis, classified as class B according to the Child-Pugh classification. 

**Table 1:** Background characteristics of the patients BMI, body mass index; CRLM, colorectal liver metastasis; ICC, intrahepatic cholangiocarcinoma; HCC, hepatocellular carcinoma

Case	Liver diseases	Age (years)	BMI	Tumor number	Tumor location	Maximum tumor diameter (mm)	Child–Pugh Classification
1	CRLM	46	22.2	1	S8	23	A
2	HCC	76	20.0	1	S8	30	A
3	ICC	58	29.6	1	S5	80	A
4	HCC	64	22.6	1	S5	31	B
5	CRLM	66	29.0	1	S8	42	A
6	CRLM	25	18.5	3	S8, S5, S4	14	A
Median (range)		61 (25–76)	22.4 (18.5–29.6)	1		30 (14–80)	

Table 2 shows the surgical procedures and outcomes of the patient cohort. Among the six cases of LLR, systematic resection was performed in four patients (66.6%), with a median difficulty score of 7.5 (intermediate level) [10]. No conversions to open surgery were observed. The median operative time and estimated blood loss were 433 minutes (range, 337-597 min) and 178.5 mL (range, 100-1000 mL), respectively.

**Table 2:** Surgical procedures and outcomes of the enrolled patients COVID-19: coronavirus disease 2019

Case	Difficulty Score	Surgical procedure	Estimated blood loss (mL)	Operative time (min)	Surgical margin (mm)	Clavien–Dindo classification	Postoperative complication	Postoperative hospital stay (days)
1	8	S8 segmentectomy	350	555	7	0	none	12
2	9	S8 segmentectomy	200	444	1	0	none	12
3	7	S4a+S5 segmentectomy	1000	597	3	2	Bile leak	18
4	6	S5 partial resection	157	357	7	1	COVID-19 infection	20
5	6	S8 partial resection	100	337	10	0	none	8
6	10	S5 segmentectomy + S8 partial resectionx2 + S4 partial resection	103	422	5	0	none	8
Median (range)	7.5 (6–10)		178.5 (100–1000)	433 (337–597)	6 (1–10)			12 (8–20)

Postoperative complications (Clavien-Dindo classification grade II or higher) were observed in one case, which was a bile leak detected intraoperatively. In this case, a C-tube was placed intraoperatively, and the drain was removed without any postoperative complications. No deaths occurred in the study cohort. The median postoperative hospital stay was 12 days (range, 8-20 days). Negative surgical margins were achieved in all six cases.

## Discussion

An improved understanding of the complex anatomy of the liver can be achieved by collecting 3D information. Although conventional techniques allow for confirmation only in two dimensions, current advancements in VR and MR have made it possible to comprehend the structure of the liver in 3D. Both technologies have made remarkable progress and are used not only in video games but also in everyday life, including industries such as construction and manufacturing. Reports of the application of VR are gradually increasing in medicine [13,14]. However, there are few studies on the application of VR and MR in LLR [15,16]. Here, we described the application of LLR using XR technology, which specifically combined VR and MR technologies, to enhance surgical visualization and anatomical recognition.

Obtaining an accurate stereoscopic space within the liver is a technical challenge of LLR [17]. Intraoperative ultrasound is commonly used during surgery; however, if it were possible to easily obtain realistic images, this could supplement anatomical understanding. One of the main advantages of XR technology is its ability to recreate and visualize 3D models based on preoperative imaging data, and in recent years, this approach has become increasingly common. The service provided by Holoeyes MD, which we used in this study, allows for easy creation of holograms using these 3D images with minimal hindrance. By converting the 3D data into polygon models, we generated holograms that provided stereoscopic vision and a virtual environment in real time. This allowed for a comprehensive understanding of liver anatomy prior to surgery, including potential exposure of the Glisson and vein branches.

Interactive manipulation in the VR environment enabled intuitive confirmation of the positioning and movement of the liver in its actual passive state. This preoperative VR visualization aided in surgical planning and decision-making, enhancing the precision and safety of the procedure. During surgery, the use of HoloLens2 MR technology further improved the surgical experience. Wearing the HoloLens2 device enabled the surgeon to view holograms overlaid on the field of vision, providing real-time guidance and visualization during dissection. In the past, surgeons required some time to learn the method of operating the HoloLens with this technology; however, this was reported to improve during the procedure [18]. In this study, intraoperative bile leakage was confirmed in one patient; however, it was caused by a technical error that damaged the exposed Gleason and was not due to non-recognition of the anatomy. In the other cases, despite a high difficulty score, there were no major complications, which may have contributed to the improvement in safety. Additionally, improved visual information may have also had an impact on robotic surgery, where tactile sensation is said to be lacking. Combining AR, VR, and MR with robot-assisted surgery has the potential to reduce surgeon fatigue and has been reported to increase the accuracy of spinal realignment and stabilization [5].

However, the requirements of specialized equipment must be considered. The use of XR technologies, such as VR and MR, necessitates the availability of specific devices and tools. In this study, the HoloLens2 was used, which is a head-mounted display device. However, as it is based on preoperative thin-cut images, it is difficult to incorporate XR technology into surgery in situations where imaging studies are inadequate. Furthermore, the practicality of XR technology in surgical settings may be hindered by battery-related challenges. XR devices, such as HoloLens2, require a power source to function, and continuous use during lengthy procedures may pose difficulties owing to battery life. Unfortunately, since the HoloLens 2 relies on its battery, there are no solutions available for the battery capacity itself. As a countermeasure on the user side, it is realistic to use the device when necessary, such as before and after Glisson and hepatic vein treatment, and turn off the power when it is not needed.

There are limitations to consider in this study. This report is a retrospective single-center report, and the number of cases is still small, making it difficult to perform a statistical examination. The XR technology is in its early stages of application in laparoscopic liver surgery. Therefore, further investigation is required to examine the actual efficacy. Additional research and development are needed to optimize the application of this technology and explore its full potential. Although there have been indications of its usefulness, the number of studies are few [10-14]. Previous studies have shown that the combination of XR technology with laparoscopic cholecystectomy performed by mid-career surgeons contributed to a decrease in blood loss and complication rates [19]. The improved understanding of complex anatomy could efficiently enhance the learning curve, potentially speeding up the skill development of surgeons from junior to mid-career. A large-scale study analyzing the results of XR-assisted LLR versus conventional techniques, including not only surgical outcomes but also educational approaches, is valuable in assessing the long-term advantages and limitations of this technique.

## Conclusions

The integration of XR technology into LLR offers enhanced visualization, precise anatomical recognition, and real-time guidance during surgical procedures. The potential of XR technology in LLR lies not only in preoperative planning but also in intraoperative decision-making, leading to improved surgical outcomes and enhanced patient safety. As XR technology continues to evolve, further applications in minimally invasive liver surgery, including non-haptic robotic surgery and other surgical fields, are expected in the future, potentially leading to a revolution in surgical practice.
